# Single-center prospective study on thyroid function outcomes and neurological prognoses at 3 years of age in children with mild neonatal hyperthyrotropinemia

**DOI:** 10.3389/fendo.2025.1548086

**Published:** 2025-07-03

**Authors:** Guoyu Sun, Rui Zhang, Jianing Zhang, Yanxia Zhou, Zezhong Tang, Lili Liu, Xinlin Hou

**Affiliations:** Department of Pediatrics, Peking University First Hospital, Beijing, China

**Keywords:** neonates, hyperthyrotropinaemia, hypothyroidism, levothyroxine, neurological prognoses

## Abstract

**Objective:**

To explore early management strategies for full-term neonates with TSH 5–10 mU/L and normal FT4.

**Methods:**

In this single-center longitudinal prospective study, 88 neonates diagnosed at 7–14 days were followed to age three.

**Results:**

94.3% (83/88) had transient TSH elevation; 77 normalized within two months without treatment, while six received levothyroxine (3–5µg/kg/day). Five neonates (5.7%) exhibited persistent hyperthyrotropinemia and had significantly higher initial TSH. No hyperthyroidism was observed. Following up to 3 years old, only one child (1/81, 1.2%) exhibited development delay in personal-social development. Neonates with transient hyperthyrotropinaemia scored higher in problem-solving and personal–social domains than those with persistent hyperthyrotropinaemia. Neonatal FT4 at 7–14 days, timing of TSH normalization, and maternal early-pregnancy FT4 influenced the neurodevelopment of neonates. Infants of mothers with gestational diabetes scored lower in the personal–social domain.

**Conclusions:**

Persistent hyperthyrotropinemia occurs in 5.7% of mild cases and is associated with higher initial TSH. Levothyroxine at 3–5 µg/kg/day is both adequate and safe. The majority of neonates got a normal neurodevelopment by age 3, and the subtle difference between transient and persistent hyperthyrotropinemia was impacted by both maternal and neonatal factors.

## 1 Introduction

Hyperthyrotropinaemia, also known as subclinical hypothyroidism, is characterized by elevated thyroid stimulating hormone (TSH) levels without decrease in free thyroxine (FT4) levels (1). The reported incidence of hyperthyrotropinaemia in neonates varies from 0.001% to 0.1%, because of the varying testing age and the definition of the lower boundary for elevated TSH levels (2). However, studies at our medical center on neonates born to mothers with thyroid disorders during pregnancy have identified that high TSH levels are not uncommon at 7 days after birth, with over a hundred cases annually. It is also common for neonates who have mildly elevated serum TSH levels (5–10 mU/L) by 1 month after birth.

Most neonates with hyperthyrotropinaemia turned out to be normal in few months without treatment and resulted in a good neurodevelopment. However, approximately 9% of neonates with mildly elevated TSH levels (5 mU/L< TSH ≤ 10 mU/L) within the first month after birth later develop congenital hypothyroidism (CH) (3). Since the developing brain critically depends on thyroid hormones during the first 2–3 years (2), it’s important to recognize infants who need to be treated. While, affected by multiple factors, whether the hyperthyrotropinaemia was transient or persistent or resulting in CH, it’s hard to predict.

Currently, guidelines are inconsistent regarding whether treatment is necessary for these neonates. The European guidelines (1) on congenital hypothyroidism recommend proactive treatment, while the American guidelines (4) suggest that the decision on whether to initiate treatment should be made by a pediatric endocrinologist. In contrast, the Japanese guidelines (5) are more conservative. The consensus on congenital hypothyroidism in China (6) notes that there is still controversy regarding the management of infants whose TSH levels consistently remain between 6 mU/L and 10 mU/L. So, clinicians often tend to prescribe medicine to prevent the adverse effects of hypothyroidism on infants and young children.

The neurological prognosis of neonatal hyperthyrotropinaemia is contradictory. A systematic review showed that none of the 94 neonates with isolated hyperthyrotropinaemia (normal FT4 level) from nine studies had adverse developmental outcomes when excluding cases diagnosed of deafness and Down syndrome. However, 82% (77/94) cases received levothyroxine (LT4) treatment (2). A population-based record-linkage study from 1994 to 2008 in New South Wales, showed that with the increase of TSH concentration above 75th percentile, children scored less than the national minimum standard for numeracy (7). It may because that higher TSH concentration is associated with a high risk of CH and lower thyroid hormone impairs cognition. Besides, TSH promots the proliferation of Agrp1 neurons that may relate to gradual neurodegeneration, and impaired cognitive and motor functioning (8).

We conducted this prospective observational study on full-term neonates with serum TSH levels between 5 mU/L and 10 mU/L and normal FT4 levels at 7–14 days after birth at our medical Center. These children were managed and followed up with regular monitoring of thyroid function, and a neurodevelopmental assessment was conducted at 3 years of age. Through this, we aimed to explore early clinical management strategies for mild hyperthyrotropinaemia by summarizing the thyroid function outcomes and neurological prognoses of these neonates at 3 years of age.

## 2 Materials and methods

This was a prospective, observational study carried on Peking University First Hospital from January 2019 to December 2019.

### 2.1 Participants

Maternal thyroid hormones, related antibodies, and antithyroid medications can affect fetal thyroid function, potentially leading to thyroid function abnormalities in the fetus or neonate. Currently, for neonates whose mothers have thyroid structural diseases or functional abnormalities, our hospital conducts venous serum thyroid function tests (TSH, triiodothyronine [T3], free triiodothyronine [FT3], thyroxine [T4], FT4) 7 to 14 days after birth, with parental permission.

The inclusion criteria included: neonates with a gestational age (GA) of 37 weeks to 41^+6^ weeks; serum thyroid function tests completed 7 to 14 days after birth, with TSH levels between 5 mU/L and 10 mU/L, without a decrease in FT4; neonates who were followed up till 3 years of age, with developmental assessments through the Ages and Stages Questionnaire (ASQ)-3 and Ages and Stages Questionnaire-Social Emotional (ASQ-SE) scales; mothers who had regular antenatal check-ups and thyroid function monitoring at our hospital during pregnancy; and those whose parents or guardians provided informed consent.

The exclusion criteria included: small for gestational age (SGA); diagnosis of brain injury-related conditions (including but not limited to neonatal hypoxic-ischemic encephalopathy, grade III-IV periventricular-intraventricular hemorrhage, hypoglycemic encephalopathy, bacterial meningitis, and metabolic encephalopathy) or presence of high-risk factors for brain injury or development (including but not limited to neonatal asphyxia, neonatal sepsis, severe hyperbilirubinemia, and chromosomal disorders).

### 2.2 Research methods

#### 2.2.1 Thyroid-related monitoring and interventions

**Figure 1:**
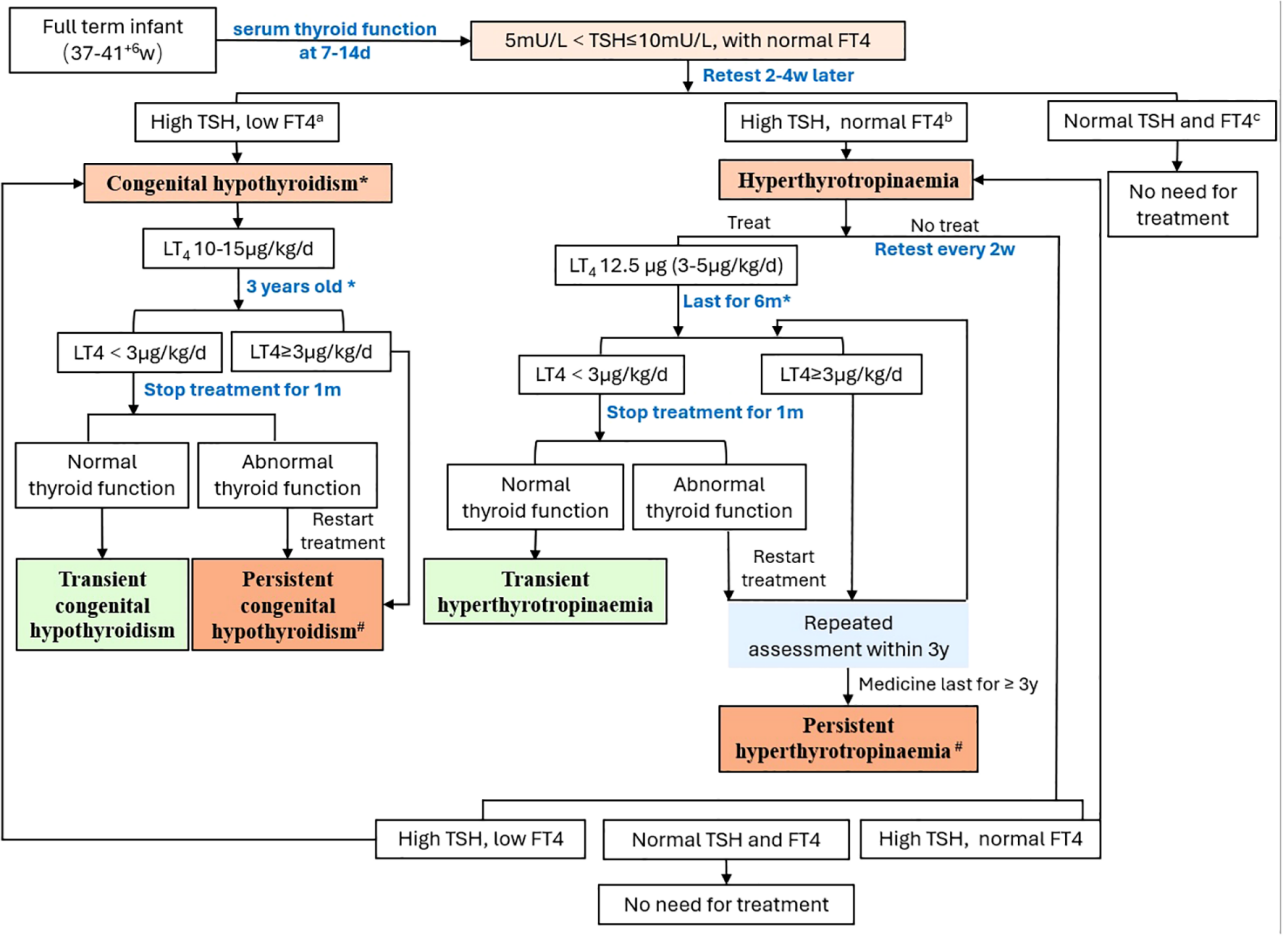
The management flowchart of mild neonatal hyperthyrotropinaemia.

Neonates underwent their first serum thyroid function test between 7 and14 days after birth. For neonates with TSH levels between 5 mU/L and 10 mU/L without a decrease in FT4, a serum thyroid function test was repeated 2–4 weeks later. If the TSH levels were elevated with a concurrent decrease in FT4, LT4 was administered orally at 10-15 μg/kg/day, and management followed the protocol for congenital hypothyroidism. If the TSH level was greater than 5 mU/L with normal FT4 levels, and after discussion with the parents, those who did not receive medication were regularly monitored for thyroid function until normalization. For those who opted for medication, LT4 was administered orally at 12.5 μg per day (approximately 3-5 μg/kg/day). Decisions on whether to adjust the medication dose were made on the basis of subsequent thyroid function test results, with thyroid function tests repeated 4 weeks after each dose adjustment. If the LT4 dose was less than 3 μg/kg/day after 6 months, discontinuation of the medication could be attempted. Thyroid function tests were repeated 4 weeks after discontinuation, and if the results were normal, the discontinuation was continued under observation. If TSH levels increased again, patients resumed taking LT4. Those receiving medication for more than 6 months required a thyroid ultrasound. At 3 years of age, if the LT4 dose was less than 3 μg/kg/day and the repeated thyroid ultrasound was normal, an attempt to discontinue the medication could be made again. Four weeks after discontinuing the medication, thyroid function tests were repeated. If the results were normal, the observation without medication continued. However, if thyroid function became abnormal again after discontinuing the medication, it was recommended that the parents and child undergo whole exome sequencing (WES) and copy number variation (CNV) analysis. Further decisions on medication were based on the thyroid function test results. Those who discontinued medication and had normal thyroid function within the first 3 years after birth were defined as having transient hyperthyrotropinaemia. Those who experienced elevated TSH levels again after discontinuing medication at 3 years of age were defined as having persistent hyperthyrotropinaemia (2) (**Figure 1**).

#### 2.2.2 Neonatal neurodevelopmental assessment

The ASQ demonstrates good consistency with the Bayley Scales of Infant Development in terms of sensitivity and specificity (9). Therefore, in this study, professional medical staff assisted parents in completing the ASQ assessment when the infants reached 3 years of age. The ASQ consists of two parts: ASQ-3 and ASQ-SE (10). The ASQ-3 focuses on five developmental domains: communication, gross motor, fine motor, problem solving, and personal-social. Infant who scored higher than the cutoff was normal; individual who scored near the cutoff was borderline; individual who scored below the cutoff was considered developmentally abnormal. The ASQ-SE is specifically designed to assess social-emotional development. A total score equal to or above the cutoff indicated that the infant was at risk for social-emotional disorders. If the ASQ-3 score was below the cutoff in any developmental domain, or if the ASQ-SE results were abnormal, the outcomes were interpreted as abnormal.

### 2.3 Statistical analysis

Statistical analyses were performed using SPSS software version 22.0 (Armonk, NY: IBM Corp). The Kolmogorov–Smirnov test was used to determine whether the parameters followed a normal distribution. Normally distributed continuous data are expressed as means ± standard deviations ( $\bar{x}$ ± s), with intergroup comparisons conducted using the independent sample t-test. Categorical data are expressed as cases (%), with intergroup comparisons conducted using the chi-squared or Fisher’s exact test. Pearson correlation analysis and multiple linear regression models were used to analyze factors influencing early postnatal TSH elevation. Receiver operating characteristic (ROC) curves were used to explore serum TSH thresholds predicting persistent hyperthyrotropinaemia. A p-value of less than 0.05 was considered statistically significant.

### 2.4 Ethical approval statement

This study was reviewed and approved by the Clinical Research Ethics Committee of Peking University First Hospital (approval number: 2018Y255).

## 3 Results

### 3.1 Demographic data

The study included a total of 88 neonates from January 2019 to December 2019, comprising 56 males and 32 females, resulting in a male-to-female ratio of 1.7:1. The average GA was 39.3 ± 1.1 weeks, and the average birth weight was 3364 ± 478g. The first serum thyroid function test was conducted at 9 ± 2 days after birth (range: 7–14 days). The average TSH level was 7.1 ± 1.4 mU/L, and the FT4 level was 20.5 ± 3.5 pmol/L. The mothers had an average age of 32 ± 4 years (range: 24–44 years) and all had a university education. Among the mothers, 80 had hypothyroidism or subclinical hypothyroidism, four had hyperthyroidism (with TR-Ab positive in two mothers and TPO antibody positive in the other two), and four had thyroid nodules without thyroid function abnormalities. TPO antibody was positive in 56 mothers while negative in the other 32. Additionally, 18 mothers had comorbid gestational diabetes mellitus and seven had comorbid gestational hypertension or preeclampsia.

### 3.2 Factors influencing mild neonatal hyperthyrotropinaemia

**Figure 2:**
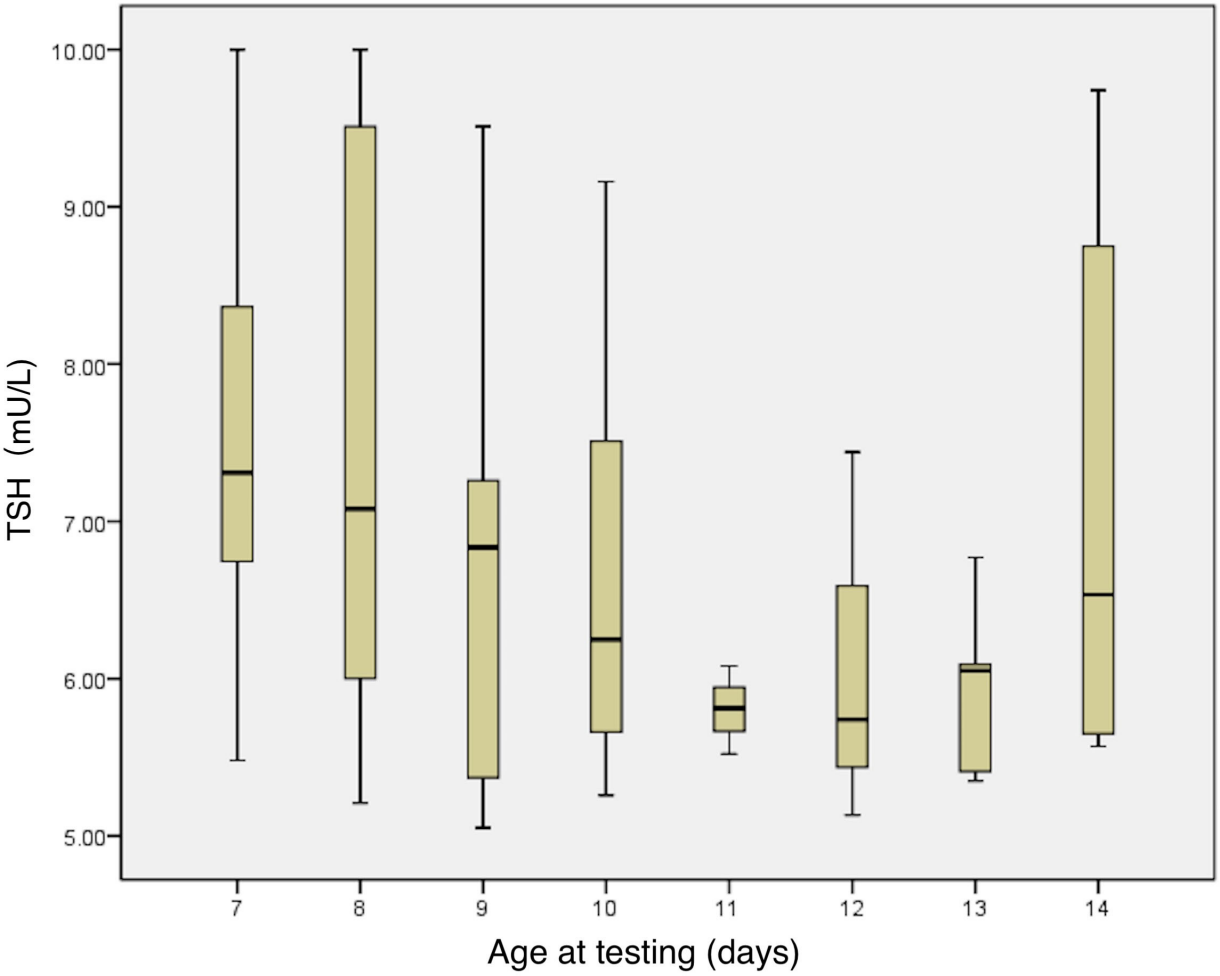
Relationship between serum TSH levels and age in days at the first thyroid function test. TSH, thyroid stimulating hormone.

This study analyzed the relationship between neonatal serum TSH levels at 7–14 days after birth and factors such as sex, GA, birth weight, age at the first serum thyroid function test, maternal age, maternal TSH and FT4 levels in early/late pregnancy, whether the mother took thyroid-related medications (LT4 and antithyroid medications), whether the TPO antibody was positive during pregnancy, and whether the mother had gestational diabetes mellitus or gestational hypertension/preeclampsia. The correlation analysis demonstrated: GA, birth weight, and age at the first serum thyroid function test were somewhat correlated with TSH levels (r= -0.249; *p*=0.019; r= -0.219; *p*=0.04; and r= -0.282; *p*=0.008, respectively); neonates born to TPO-antibody positive mothers had lower serum TSH levels (6.60 ± 1.22mU/L *vs*. 7.31 ± 1.49mU/L, *p*=0.025) and higher FT4 (21.25 ± 3.44mU/L *vs*. 20.00 ± 3.52mU/L, *p*=0.111); neonates born to mothers with gestational diabetes mellitus had higher TSH levels compared with those born to mothers without gestational diabetes mellitus (7.8 ± 1.6 mU/L *vs*. 6.9 ± 1.3 mU/L, respectively; *p*=0.018). A multiple linear regression analysis showed that the regression equation was significant (F=4.906, *p*=0.001). Younger age at the first serum TSH test (β = -0.266, *p*=0.008, **Figure 2**) and mother with negative TPO antibody (β = -0.257, *p*=0.01) are related to higher TSH levels.

### 3.3 Thyroid function outcomes of mild neonatal hyperthyrotropinaemia

**Figure 3:**
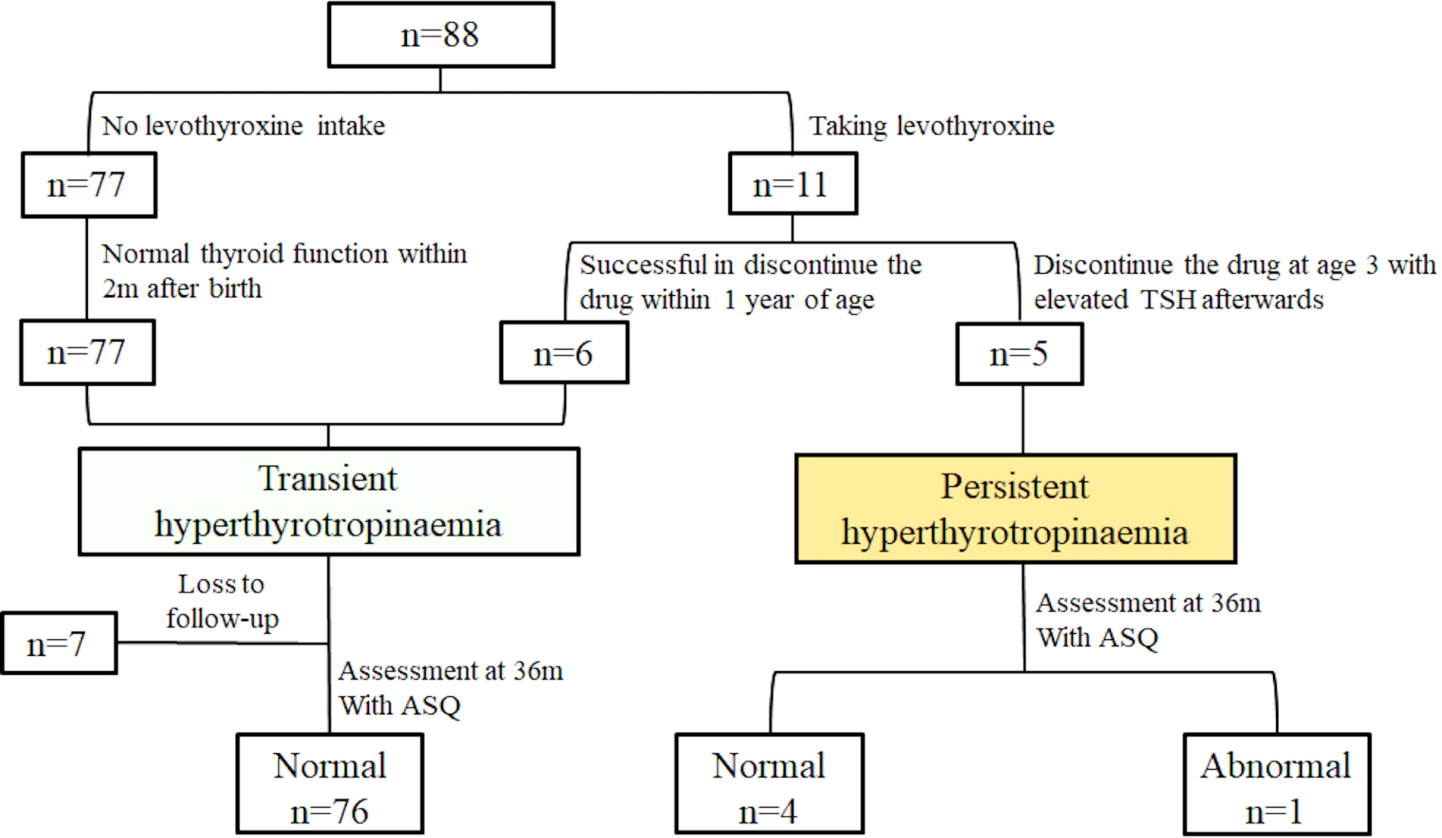
Thyroid function outcomes and neurological prognoses at 3 years of age in the 88 neonates with hyperthyrotropinaemia.

Among the 88 neonates, 77 (87.5%) did not require medication and their thyroid function normalized within 31.1 ± 11.2 days (range: 20–60 days), indicating transient hyperthyrotropinaemia. However, 11 (12.5%) had serum TSH levels remaining above 5 mU/L at 21 days after birth and began taking LT4. The age at the start of medication was 47 ± 19 days (range: 23–82 days) after birth, with an initial dose of 12.5 μg/day (3-5 μg/kg/day). Thyroid function tests repeated 2–4 weeks later showed normalization. Six infants successfully discontinued medication within the first year (4–10 months) after birth and had normal thyroid function at 3 years of age, indicating transient hyperthyrotropinaemia. However, five (5.7%, 5/88) exhibited persistent hyperthyrotropinaemia (**Figure 3**).

Of the five children with persistent hyperthyrotropinaemia, four were unable to discontinue LT4 before 3 years of age, with two requiring increased LT4 doses of 25-37.5 μg/day (3-5 μg/kg/day) due to recurrent TSH elevation. One boy discontinued LT4 treatment at 4 months of age, and during follow-up until the age of 3 years, his serum TSH level was 6.5 mU/L and FT4 was 15.2 pmol/L. Thyroid ultrasounds showed no abnormalities in any of the children included in the study. One child with persistent hyperthyrotropinaemia underwent WES testing, which revealed a heterozygous *TSHR* variant (AD, c.1349(exon10)G>A, p.R450H(p.Arg450His)), a missense mutation inherited from the mother. The mother was found to have subclinical hypothyroidism while preparing for pregnancy; hence, she took LT4 and her thyroid function normalized. The child’s younger sister, currently 1 year and 8 months old, also presented with hyperthyrotropinaemia during her neonatal period. She was given a small dose of LT4 (12.5 μg/day), which helped her maintain normal thyroid function. Her thyroid ultrasound was normal and her genotype matched her mother and brother. According to American College of Medical Genetics and Genomics guidelines, this genetic variant is considered potentially pathogenic.

**Figure 4:**
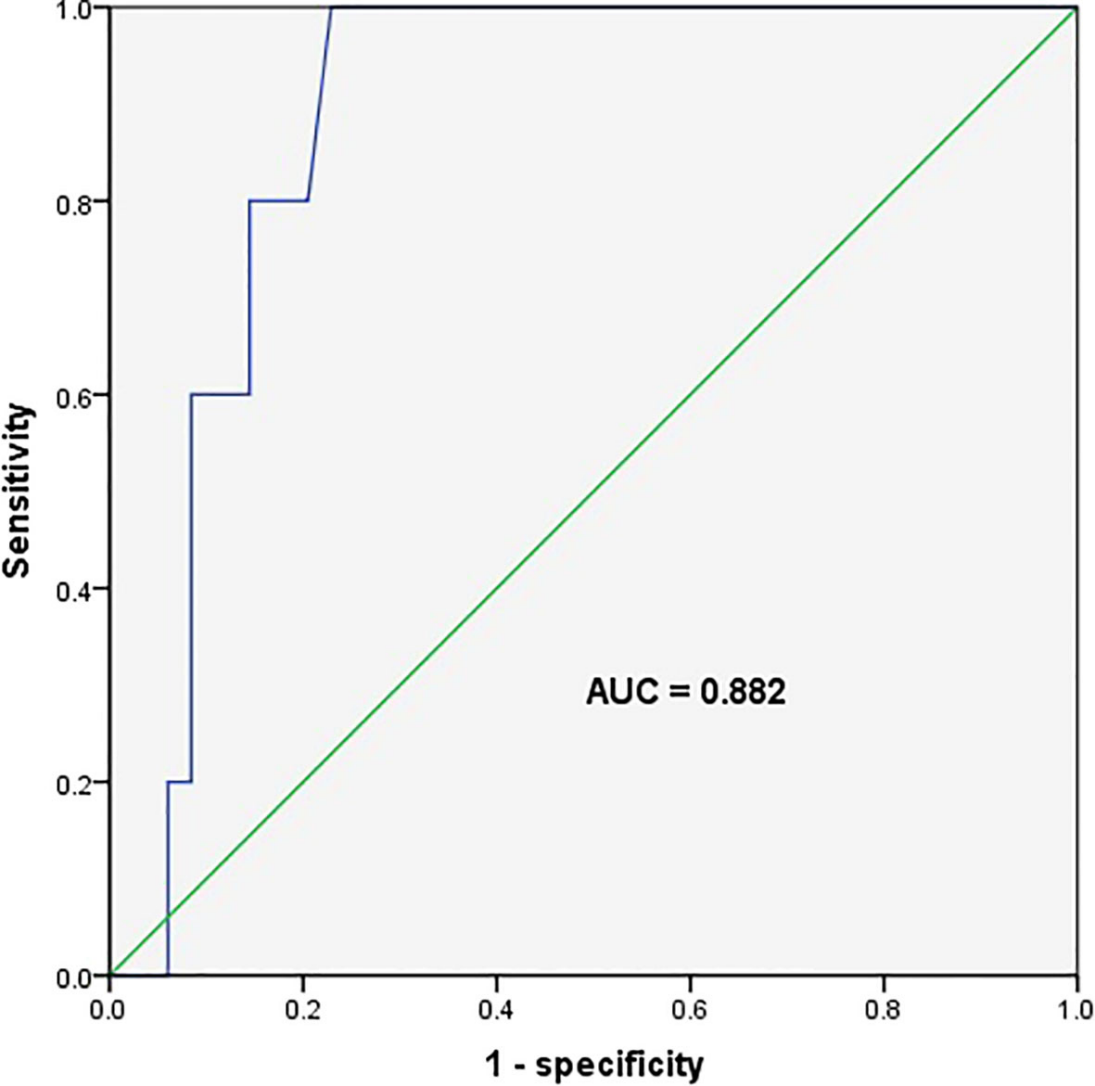
Receiver operating characteristic curve for the initial serum thyroid stimulating hormone level predicting persistent hyperthyrotropinaemia. The area under the ROC curve (AUC) was 0.882 (95% CI, 0.803–0.960), with a sensitivity of 100% and a specificity of 77.1% at the cut-off point (7.67 mIU/L).

Compared with transient hyperthyrotropinaemia, the initial TSH levels in the five children with persistent hyperthyrotropinaemia were higher (9.0 ± 0.8 mU/L *vs*. 6.9 ± 1.4 mU/L, respectively; *p*=0.001). The ROC curve indicated that the initial serum TSH level could serve as a marker to distinguish between persistent and transient hyperthyrotropinaemia, with an area under the curve of 0.882 and a 95% confidence interval of 0.803 to 0.960 (*p*=0.004). The maximum Youden index corresponded to a threshold of 7.67 mU/L (sensitivity 1.000; specificity 0.771), indicating that this level could serve as a cut-off point to distinguish between persistent and transient hyperthyrotropinaemia (**Figure 4**).

### 3.4 Neurodevelopmental outcomes at 3 years of age in children with mild neonatal hyperthyrotropinaemia and factors influencing the prognosis

Among the 88 neonates, 81 (92%) were followed up and completed the ASQ assessment at 3 years of age. Of these 81, 76 had normal development and four scored near the cutoff. Specifically, two showed lower scores in gross motor skills, one in problem solving and one in personal-social domains. Subsequent assessments using the Griffiths Scales of Child Development showed normal results. Only one child (1/81, 1.2%) showed developmental abnormality, specifically lagging behind peers in personal-social domain. A comparison of the five development domains between children with transient and persistent hyperthyrotropinaemia showed that those with transient hyperthyrotropinaemia scored significantly higher in the domains of problem solving (57.2 ± 4.4 *vs*. 51.0 ± 5.5, respectively; *p*=0.003) and personal-social (56.5 ± 4.0 *vs*. 51.0 ± 12.5, respectively; *p*=0.016) at 3 years of age (**Table 1**).

**Table 1:** Ages and Stages Questionnaire assessment results at 3 years of age in neonates with transient *vs* persistent hyperthyrotropinaemia.

ASQ-3/ASQ-SE	Transient hyperthyrotropinaemia	Persistent hyperthyrotropinaemia	p-value
Communication	58.0 ± 3.0	58.0 ± 2.7	0.977
Gross motor	56.5 ± 4.0	55.0 ± 3.5	0.413
Fine motor	52.2 ± 5.6	48.0 ± 7.6	0.119
Problem solving	57.2 ± 4.4	51.0 ± 5.5	0.003
Personal-social	56.5 ± 4.0	51.0 ± 12.5	0.016
Social-emotional	29.5 ± 12.1	20.0 ± 10.6	0.092

This study further analyzed the relationship between neurodevelopmental outcomes at 3 years of age and various factors, including sex, GA, birth weight, serum TSH and FT4 levels at 7–14 days after birth, age at which thyroid function normalized, maternal age, maternal TSH and FT4 levels during early and late pregnancy, and whether the mother had gestational diabetes mellitus. The results showed that: serum FT4 levels at 7–14 days after birth were positively correlated with scores in the domain of fine motor (r=0.224, *p*=0.044); the older the age at which neonatal thyroid function normalized, the lower the scores in the domain of problem solving (r=-0.280, *p*=0.011); abnormal maternal FT4 levels during early pregnancy also led to lower scores in the problem solving (57.3 ± 3.9 *vs* 50 ± 13.2, respectively; *p*=0.006); neonates born to mothers with gestational diabetes mellitus scored lower in the personal-social domain at 3 years of age compared with those born to mothers without gestational diabetes mellitus (56.7 ± 4.0 *vs*. 53.8 ± 7.4, respectively; *p*=0.032).

## 4 Discussion

This study indicates that mild hyperthyrotropinaemia was relatively common among neonates at our medical center, with a higher incidence than previously reported in the literature (2). This may be attributed to the inclusion of neonates born to mothers with thyroid disorders during pregnancy. Our study showed that 94.3% (83/88) of the neonates had transient hyperthyrotropinaemia, with thyroid function normalizing as their age in days increased. However, 5.7% (5/88) of the neonates still exhibited persistent hyperthyrotropinaemia. At 3 years of age, 98.8% (80/81) of the neonates with mild hyperthyrotropinaemia exhibited normal neurodevelopment. However, at 3 years of age, children with persistent hyperthyrotropinaemia scored lower in problem solving and personal-social domains compared with those with transient hyperthyrotropinaemia. This suggests the need for long-term follow-up of thyroid function and neurodevelopment in children with persistent hyperthyrotropinaemia.

Flávia’s study (3) showed that neonates with TSH levels between 5–10 mU/L in newborn screening, 9.13% subsequently developed congenital hypothyroidism and could not discontinue LT4 treatment within the first 2 years after birth. However, in this study, 94.3% of the neonates had transient hyperthyrotropinaemia, lower than reported and no cases of congenital hypothyroidism were identified. It might be due to the difference in regions and crowds. Mothers in this study all suffered from thyroid-related disorders during pregnancy. As known, neonatal thyroid function is influenced by maternal thyroid hormone levels during pregnancy, thyroid-related antibodies, and relevant medications (such as LT4 and antithyroid medications) (11). Thyroid-related medications may be metabolized within the first month after birth (antithyroid medications metabolize faster), while thyroid-related antibodies can persist in the neonate’s body for 1–3 months. It explained why the abnormal thyroid function in transient hyperthyrotropinaemia of our study lasted for more than one month, till 2–3 months later without treatment. Interestingly, most studies regard that TPO antibody has little effect to fetus. However, our study found that neonates of mothers with positive TPO antibody had lower TSH levels and higher FT4 levels, which may due to the attack of TPO antibody to neonatal thyroid gland as Hashimoto thyroiditis and this needs to be confirmed in a large-scale study.

Current studies indicate (12–14) that the higher the TSH levels, the greater the likelihood of developing congenital hypothyroidism or persistent hyperthyrotropinaemia. The results of our study showed that neonates with persistent hyperthyrotropinaemia had significantly higher serum TSH levels within 7–14 days after birth compared with those with transient hyperthyrotropinaemia. Neonates with serum TSH levels exceeding 7.67 mU/L were at a higher risk of developing persistent hyperthyrotropinaemia, and required LT4 treatment and long-term follow-up. The most common cause of persistent hyperthyrotropinaemia is structural abnormalities in thyroid development, such as athyreosis, thyroid hypoplasia, or ectopic thyroid (2, 15). In our study, thyroid ultrasound conducted on infants with persistent hyperthyrotropinaemia did not reveal any abnormalities. Another cause that requires attention is genetic mutations, which can lead to regulatory abnormalities of the hypothalamic-pituitary-thyroid axis or directly cause impairments in the synthesis and secretion of thyroid hormones, resulting in thyroid function abnormalities. In our study, one child with persistent hyperthyrotropinaemia underwent family-based WES and CNV testing. The results found a heterozygous TSHR variant, inherited from his mother, with the child’s little sister sharing the same genotype. This gene can exhibit either autosomal recessive or dominant inheritance and shows genetic heterogeneity. In patients with heterozygous mutations, thyroid hormone levels are maintained within the normal range through a compensatory elevation of serum TSH levels, while thyroid size and iodine uptake usually remain normal (16). Homozygous mutations often result in more severe manifestations, with higher serum TSH levels; moreover, serum thyroid hormone levels remain low and there may be thyroid dysgenesis (17).

Due to the lack of large-scale controlled studies, the future developmental outcomes of infants with hyperthyrotropinaemia and the potential risks of not treating this condition remain unclear. Currently, the guidelines from various countries regarding whether these neonates need treatment are inconsistent. The guidelines on the monitoring and management of congenital hypothyroidism published by the American Academy of Pediatrics (4) state that for infants with serum TSH levels between 5 mIU/L and 10 mIU/L at 4 weeks after birth, further treatment should be determined after a pediatric endocrinologist is consulted. The guidelines from the European Society for Pediatric Endocrinology (1) indicate that for neonates older than 21 days with TSH levels between 6 mU/L and 20 mU/L and normal FT4 levels, treatment with LT4 should be initiated immediately, with an initial dose of 5-10 µg/kg/day. The Japanese guidelines on congenital hypothyroidism (5) recommend that infants under 6 months of age (who are not neonates) with TSH levels of 10 mIU/L or more and children older than 12 months with TSH levels of 5 mIU/L or more should receive LT4 treatment, with a starting medication dose of 3-5 μg/kg/day. Our study suggested that serum FT4 levels within 7–14 days after birth and the time taken for thyroid function to normalize are associated with neurological prognostic scores at 3 years of age. Therefore, early identification of children with persistent hyperthyrotropinaemia and the use of appropriate doses of LT4 to normalize thyroid function as soon as possible may be beneficial for the long-term neurodevelopmental outcomes of these neonates (18).

Following the guideline recommendations and clinical experience (1, 19, 20), we initiated treatment with a low dose of LT4 (12.5 µg/day, equivalent to 3–5 µg/kg/day) for neonates with elevated TSH levels beyond 21 days of age. Given LT4’s half-life (T_1/2_) of 7 days, it takes approximately 1 month for blood concentrations to reach a steady state. Therefore, most infants had their first thyroid function test 4 weeks after LT4 treatment was started. The intervals for subsequent repeated thyroid function tests were generally longer than those for children with congenital hypothyroidism. Typically, repeated tests were conducted every 1–3 months within the first 6 months, every 3–4 months within the first 12 months, and every 4–6 months from 1 to 3 years of age. Under this strategy, all infants in the study showed normal results in repeated thyroid function tests after medication, and no side effects of hyperthyroidism were observed. This indicates that for neonates with mild hyperthyrotropinaemia, starting LT4 treatment at a dose of 3-5 µg/kg/day may be appropriate. Moreover, appropriately extending the intervals between repeated tests reduced the economic and psychological burden on the children and their families.

Consistent with the results of this study, most current research (12, 21) indicates that the risk of adverse neurological prognoses is not increased in infants and young children with hyperthyrotropinaemia. The study by Unüvar (12) showed that there were no significant differences in the results of the Denver developmental screening test and the Wechsler intelligence scale between children with persistent and transient hyperthyrotropinaemia (TSH > 10 mU/L). However, our study found that children with persistent hyperthyrotropinaemia (5mIU/L< TSH ≤10mIU/L) scored lower in problem solving than those with transient elevations. These differences may be related to the different crowds (different TSH levels) included in the two studies. The fetal thyroid is not fully developed before 18 weeks of gestation, early fetal neurodevelopment relies predominantly on maternal thyroid hormone levels during early pregnancy (22–24). Hence, abnormal maternal FT4 levels during early pregnancy in our study, whether too low or high, affected the neurodevelopmental scores of neonates at 3 years of age. Our study also demonstrated the impact of gestational diabetes mellitus on neurodevelopmental prognosis, consistent with previous reports (25, 26).

This study had a few limitations. First, this was a single-center prospective observational study with a limited number of participants, which may lead to selection bias. In the future, multicenter clinical studies should be conducted to minimize this bias. Second, suffered by the COVID-19 pandemic, most children were unable to visit medical institutions for neurodevelopmental assessments, resulting in a decreased follow-up rate, although 92% of the children completed the ASQ developmental scale assessment by WeChat. Longer follow-up may be required to validate our findings.

## 5 Conclusion

Mild hyperthyrotropinaemia is not uncommon among neonates. In this study, 94.3% of neonates with hyperthyrotropinaemia experienced normalization of thyroid function within the first year after birth, indicating a transient elevation. Higher serum TSH levels (>7.67 mU/L) in the early neonatal period may indicate persistent hyperthyrotropinaemia, needing low-dose levothyroxine treatment. The majority of neonates got a normal neurodevelopment by 3 years of age. However, there’s difference between transient and persistent hyperthyrotropinaemia, indicating a large-scale long-term follow-up. Our study may spark further multicenter research with a larger sample size and longer follow-up period, validating our findings and helping guide the development of standardized protocols for the management of neonatal hyperthyrotropinaemia.

## Data Availability

The raw data supporting the conclusions of this article will be made available by the authors, without undue reservation.
